# Performance Comparison of Zn-Based and Al–Si Based Coating on Boron Steel in Hot Stamping

**DOI:** 10.3390/ma14227043

**Published:** 2021-11-20

**Authors:** Long Chen, Wei Chen, Miao Cao, Xin Li

**Affiliations:** 1 School of Materials Science and Engineering, Jiangsu University, Zhenjiang 212013, China; xianglongjiubian@outlook.com; 2 School of Mechanical Engineering, Jiangsu University, Zhenjiang 212013, China; 3 School of Mechanical Engineering, Xi’an Jiaotong University, Xi’an 710049, China; 4 State Key Laboratory of Materials Processing and Die and Mould Technology, Huazhong University of Science and Technology, Wuhan 430074, China; 5 China Construction Third Engineering Bureau Group Co., Ltd., Wuhan 430070, China; lixin28@cscec.com

**Keywords:** hot stamping performance, Al–Si coating, GI coating, GA coating

## Abstract

The coatings of boron steels play an important role in affecting the quality of hot stamping parts, so it is important to evaluate the hot stamping performance of coatings before designing processes. Taking the U-type hot stamping part of boron steel as research objects, the surface quality, microstructure and temperature variation of samples with GA (galvannealed), GI (galvanized) and Al–Si coatings were observed and analyzed to evaluate the anti-oxidation, forming and quenching performances of different coatings. The results show that all the GA, GI and Al–Si coatings could provide good oxidation protection and also act as the lubricants for avoiding the friction damage of sample substrates and die-surface. But the different compositions of GA, GI and Al–Si coatings will contribute the different colors. Under the same deformation degree, the Al–Si coating can provide the best substrate protection and the GI coating will induce cracks in the substrate because of the liquid metal-induced embrittlement phenomenon. There is no significant difference between the quenching performances of GA, GI and Al–Si coatings, and the thermal conductivity of the GI coating is slightly better than Al–Si and GA coatings.

## 1. Introduction

Ultra-high strength structural parts have been widely used in automotive, aircraft, buildings and so on for energy-saving and emission reduction [1]. At present, the hot stamping process is a popular technology for producing the ultra-high strength parts from boron steels [2]. The boron steel hot stamping process is as follows: the quenchable boron steel sheets (such as 22MnB5) are heated in a furnace at approximately 930 °C for several minutes to transform the sheets into austenite; subsequently, the austeniteized sheets are quickly transferred from the furnace to the stamping tool and deformed into the required shape with simultaneous rapid cooling in pressurized-closed-die; finally, the ultra-high strength part with a tensile strength of 1500 MPa is taken out from a die after the full martensite transformation of the part during cooling [3]. Actually, the surface oxidation and carbonization of the boron steel sheets during the heating process will severely reduce the final performance of hot stamping parts [4]. Thus, a series of coatings have been developed to improve the anti-oxidation properties of boron steels, especially the Al–Si coated boron steel and zinc-based coated boron steel which have been widely used for commercialized production [5,6].

The typical hot-dip Al–Si coating, used for USIBOR1500 boron steels developed by Arcelor is the eutectic Al–Si alloy that consist of 10 wt.% Si and 90 wt.% Al [7]. In the austenitization process, the Al–Si coating changes according to “solid phase → liquid phase → solid phase”, and the different heating processes will lead to the different phase transitions of Al–Si coating. After austenitization, the microcracks, originate from the coating surface and end at the diffusion zone, and this will occur in Al–Si coating even without plastic deformation due to the difference in thermal expansion coefficients of coating components [8]. When under deformation, cracks that extend vertically to the surface of the substrate occur under tensile stress and lamellar cracks appear due to compressive stress, and large tensile deformation will even cause the separation of coating and substrate. Increasing the heating temperature or extending the holding time will promote the formation of the iron-rich phase in an Al–Si coating, which will increase the ratio of ductile Fe–Al phase and then reduce the amount and depth of cracks in coating [9].

For reducing production cost and providing cathodic protection [10], the zinc-based coated boron steels, including Zn galvanized boron steel (GI coating) and Zn-Fe galvannealed boron steel (GA coating), were developed by Voestalpine in 2008 [11]. However, the formability of zinc-based coated boron steels in hot stamping process could be affected by the phenomenon of liquid metal-induced embrittlement (LMIE) [12,13]. When the sheets were formed at the temperature higher than 782 °C and, meanwhile, under tensile stress, the liquid zinc in the coating will penetrate along the austenite grain boundary and continuously react with substrate to produce α-Fe (Zn). Thus, the cracks penetrate into the substrate along the interface of α-Fe (Zn) and the liquid phase, resulting in brittle fracture of the sheets. The existence of LMIE will significantly limit the application of zinc-based coated sheets in industrial applications [7]. As known from recent research [14,15], some operations can be adopted to suppress the LMIE phenomenon, such as increasing the heating temperature or holding time, decreasing the stamping temperature (≤782 °C), and reducing the hot deformation of a coating during hot stamping.

Besides research on the defects of boron steel coating in hot stamping, research also focuses on the microstructure evolution in austenitization processes [5,7,16], corrosion resistance [17,18], oxidation resistance [18], lubrication [17,19,20] and welding [21,22]. The properties of different coating layers depend on their microstructures, and the differences in coating properties will have different influences on the forming and quenching qualities of hot stamping parts. Thus, it is necessary to conduct research on comparing the properties between different coatings, by which the applicability of each coating could be determined.

In this paper, a similar U-shaped sample with rounds, stairs and asymmetric sidewalls have been designed and the corresponding hot stamping die has also been developed. Then the anti-oxidation property, forming and quenching performances of GA, GI and Al–Si coatings were compared by observing the surface, microstructure and cooling curves of hot stamping samples obtained with GA, GI and Al–Si coatings.

## 2. Materials and Methods

### 2.1. Materials

**Figure 1:**
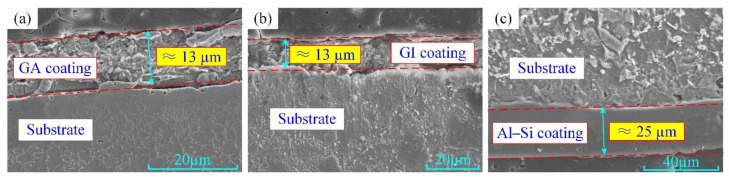
The initial state of galvannealed (GA), galvanized (GI) and Al–Si coatings: (**a**) GA coating; (**b**) GI coating; (**c**) Al–Si coating.

The hot stamping boron steel sheets B1500HS with GA, GI and Al–Si coatings were used for experiments. The chemical compositions of boron steel are listed in Table 1 [23]. The Zn-based coatings are produced by hot-dipping the boron steel into the liquid Zn-bath containing a level of Al adapted to the expected coating. Because of the different Al content in the Zn bath (GI: 0.2 wt.%, GA: 0.13~0.14 wt.%), the GI coating is full of pure zinc phase (η phase) and GA coating is the Zn-10 wt.% Fe intermetallic compound that consists of a lot of δ phase (FeZn_7_), some Γ1 phase (Fe_5_Zn_21_) and little η phase (pure zinc phase) [11]. The Al–Si coating is produced by hot-dipping the boron steel into the molten Al alloy bath with a composition of approximately 88 wt.% Al, 9 wt.% Si, and 3 wt.% Fe at about 675 °C [7]. The obtained Al–Si coating consists mainly of an aluminum matrix and pure Si (10 wt.%) formed in the eutectic reaction during the cooling of aluminizing process [8]. As shown in Figure 1, the thickness of GA and GI coating is about 13 μm and the thickness of the Al–Si coating is approximately 25 μm.

### 2.2. Samples and Devices

**Figure 2:**
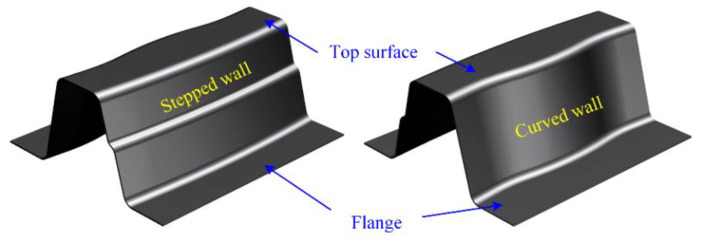
The shape characteristics of similar U-shaped sample (3D drawing).

**Figure 3:**
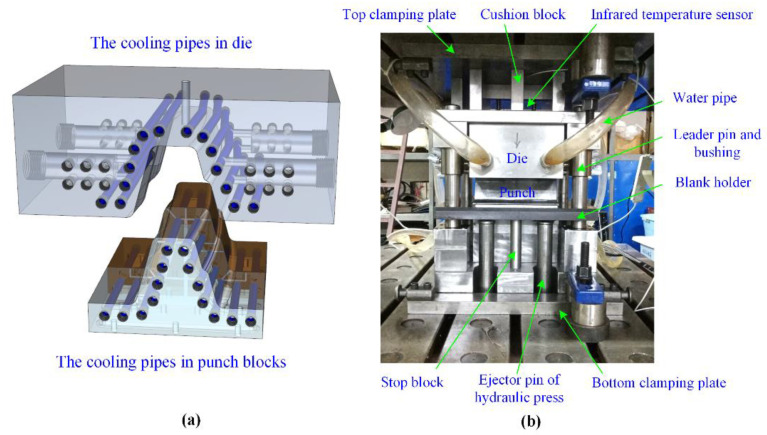
The hot stamping die for similar U-shaped sample: (**a**) the distribution of cooling pipes; (**b**) the experiment die.

The 3D drawing of the designed U-shaped hot stamping sample is shown in Figure 2, the dimension of which is 200 mm × 180 mm × 80 mm. The U-shaped sample has complex structural features, such as flange, curved surface and stepped surface, and could be the actual hot stamping part. The corresponding hot stamping experimental die is shown in Figure 3. The material of the die and punch is SDHS2 from Shanghai University and the gap value in closed die was designed as 1.4 mm. In order to minimize the wrinkles and cracking defects in forming, the blank holder force is provided by the adjustable gap between blank holder and die-surface. For guaranteeing the martensitic transformation of B1500HS in quenching, the diameter of cooling pipes is 10 mm, the distance between adjacent pipes is designed as 20~25 mm, and the minimum distance between the pipe wall and die surface is determined as 10~15 mm [24]. Moreover, the cooling pipes were evenly distributed by fitting the die-surface for the uniform quenching speed at sample everywhere. The infrared temperature sensor (Optics CT3MH1, measuring temperature range of 150~1000 °C) was used to monitor the temperature variation at the top surfaces of samples.

### 2.3. Experimental Schemes

The parameters of size and heat treatment process of boron steel sheets with Al–Si coating, GI coating and GA coating are shown in Table 2. The coated boron steel sheets were heated in the muffle furnace at 930 °C for 3~4 min, and then shifted into the die quickly. The sheets were stamped at the speed of 20 mm/s by hydraulic press and then quenched in closed water-cooling die under a load of 1000 kN. The temperature changes of samples were observed and recorded. Once the temperature of a sample decreased to 200 °C, the dies were opened to take out the samples. During the forming process, the gap between the blank holder and die-surface was 2.4 mm, which was bigger than the thickness (1.4 mm) of the sheet. Thus, the sheet at the blank holder could flow freely, which is beneficial in sheet forming.

**Figure 4:**
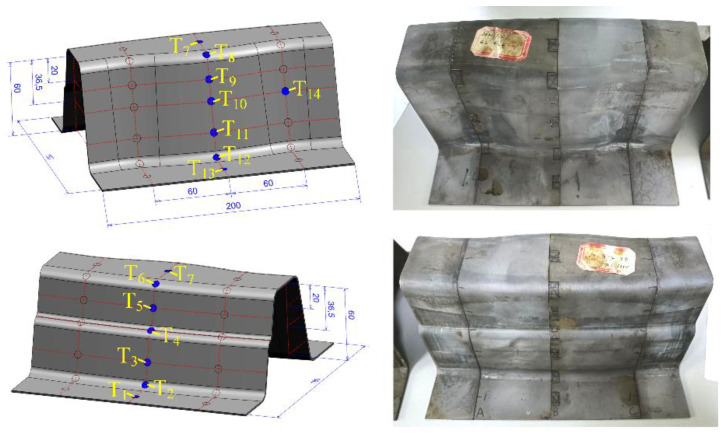
Detecting locations of the similar U-shaped hot stamping samples.

The surface qualities of hot stamping samples with GA, GI and Al–Si coatings were observed, including the shape, color, ruptures, wrinkles, and damage of the coating layers. Also, the microstructures of the similar U-shaped sample were detected. The rectangle samples (10 mm × 6 mm) were cut from the hot stamping samples at T_1_~T_14_ (see Figure 4), inlayed in epoxy resin, polished by abrasive papers, and then etched by 2% nitric acid alcohol solution. The microstructure at T_1_~T_14_ was observed by the Hitachi S-3400N scanning electron microscope. Moreover, the temperature variation curves of samples were also plotted and compared.

## 3. Results and Discussion

### 3.1. The Anti-Oxidation Properties of Coatings

**Figure 5:**
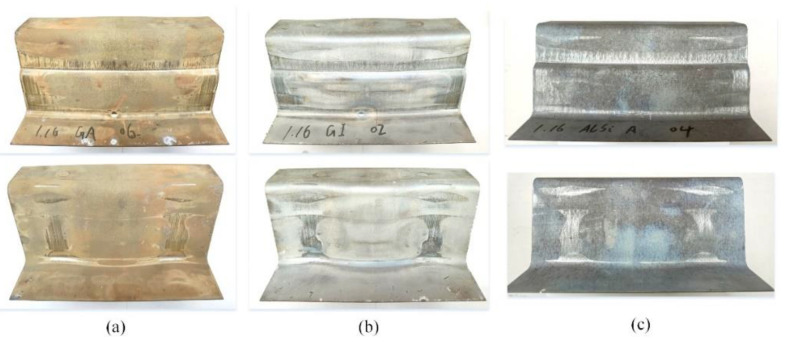
Photographs of hot stamping samples with GA coating, GI coating and Al–Si coating: (**a**) GA coating sample; (**b**) GI coating sample; (**c**) Al–Si coating sample.

The colors of as received GA, GI and Al–Si coated boron steel sheets were silver, while the hot stamped U-shaped samples exhibited different colors. The GA coated samples were a light brown, the GI coated samples were characterized by silver gray, and the Al–Si coated samples were blue gray (Figure 5).

After hot stamping, the main compositions of GA and GI coating were both α-Fe (Zn) phase and Fe_3_Zn_10_ phase [25]. The final GA coating could process a higher proportion of α-Fe (Zn) phase and a small amount of Fe_3_Zn_10_ (Г phase) than GI coating because of the higher Fe content in the original GA coating [26]. However, the final GI coating could be covered with a layer of Al_2_O_3_ oxide film because of the higher Al content in the original GI coating. As known, the original Al–Si coating was the eutectic Al–Si alloy that consist of 10 wt.% Si and 90 wt.% Al. When heated at 900~930 °C for 4 min, the Al–Si coating will mainly consist of Fe_2_SiAl_2_, Fe_5_SiAl_4_ and FeAl_2_ and be covered with a layer of Al_2_O_3_ oxide film [7]. In heating, the Fe element in sheet substrate and the oxygen element form air could diffuse into the coatings and react with the covered coating, then different oxides will form at the surface of the coating. The different Fe oxides present as different colors in visual, such as black FeO, blue Fe_3_O_4_ and red Fe_2_O_3_. The mixture of upper Fe oxides, Al_2_O_3_ and ZnO in different proportions will contribute to the different colors of the GA, GI and Al–Si coated samples in Figure 5. In summary, all the GA, GI and Al–Si coatings can prevent the oxidation of steel substrates in heating and there is no coating peeling in any hot stamping sample, showing that GA, GI and Al–Si coatings can all have a good anti-oxidation effect.

### 3.2. The Forming Performance of Different Coatings

**Figure 6:**
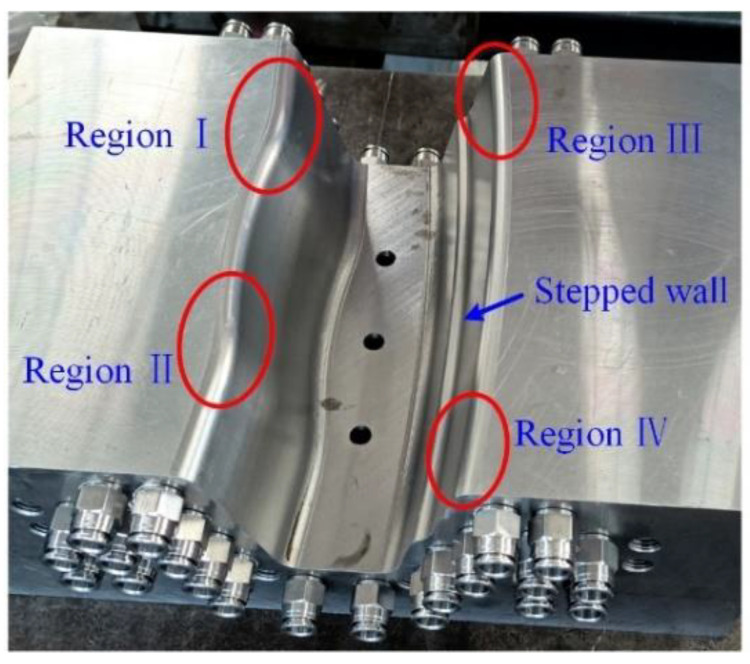
The photos of die surfaces.

**Figure 7:**
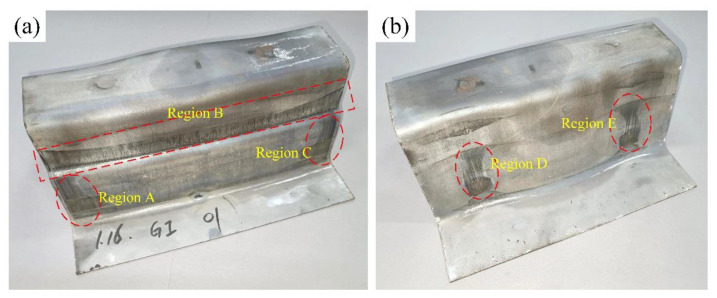
The photos of GI coated sample: (**a**) the stepped wall of sample; (**b**) the smooth wall of the sample.

As shown in Figure 5, there are no cracks and wrinkles in all hot stamping samples except some obvious wear traces. The wear of coating come from the relative sliding between sheet and die, so the forming process of sheet metal should be analyzed. During the forming of samples, the die firstly moves down until in contact with the blank holder and then they move down together when clamping the sheet. Meanwhile, the heated sheet and punch surface are gradually fitted. The sheet firstly contact the top surface of punch, followed by the rounded corners of die in region I, II, III and IV (see Figure 6) [27]. In die closing, there is a long relative sliding between the sheet and die-surface at region I, II, III and IV, where the sheet suffers high pressure because of the combined action of stretch and friction. Moreover, the coatings under high temperature are soft and easy to deform. Thus, the coatings in region A, C, D, and E (see Figure 7) have suffered significant sliding friction damage because of the poor dry frictional conditions in regions I, II, III and IV. There is also a significant extrusion trace in Figure 6 besides the wear traces. It can be seen in region B which located in the stepped side wall of U-shape sample. As die closing, the material at the top of samples firstly deforms, and then the material flows and gathers along the side wall. Finally, the sheet material in region B is extruded and the clear surface extrusion boundary is formed. Therefore, all the GA, GI and Al–Si coatings could be regarded as the lubricants and protective layers for avoiding the damage from friction and extrusion to the die-surface and sheet substrates. However, their performances in lubrication and protecting substrate are different.

**Figure 8:**
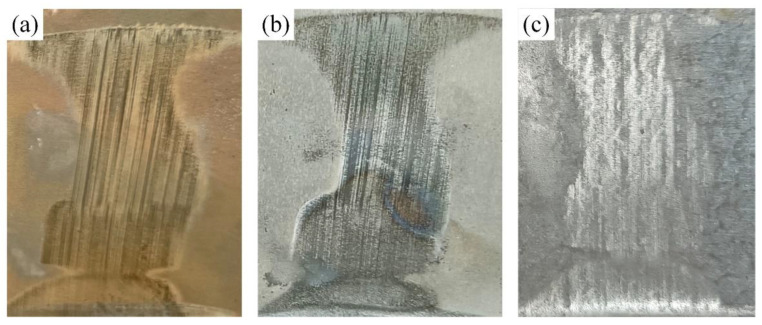
Enlarged views of coatings in region E: (**a**) region E in GA coating; (**b**) region E in GI coating; (**c**) region E in Al–Si coating.

Figure 8 shows the enlarged views of the region E in the samples obtained, where significant abrasions could be observed. It was found that the region E of GA and GI coated samples were full of tiny scratches, and the region E of the Al–Si coated sample seems to be polished rather than worn.

**Figure 9:**
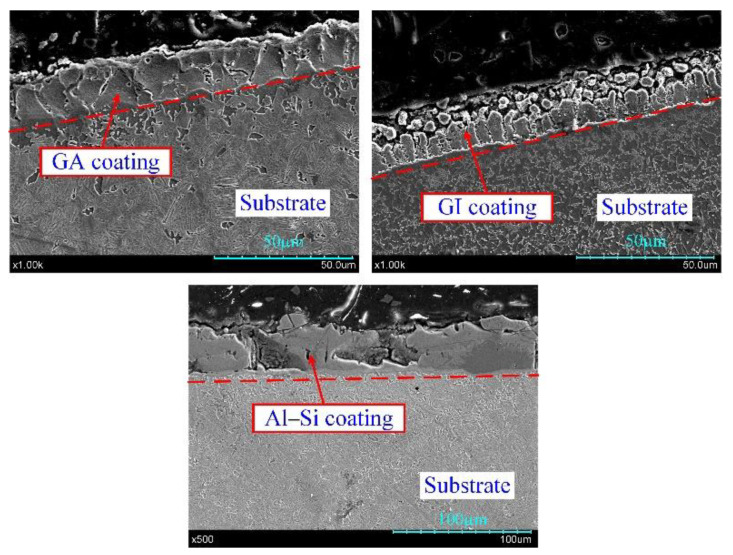
The microstructure of coatings at T_14_ in region E.

Then, the microstructures of coatings in region E (at T_14_) were analyzed by a Japan, Tokyo, Hitachi S-3400N scanning electron microscope (SEM) and listed in Figure 9. The GA coating is full of the branch-shaped cracks that extend from the coating surface to the substrate. The microstructure of the GI coating presents as two layers, the layer close to the coating surface consist of granule with different shapes and sizes, and the layer close to the substrate presents as many parallel bars perpendicular to the coating surface. A lot of microcracks in GA and GI coatings contribute to the macroscopic scratches in Figure 8a,b. There are almost no microcracks in Al–Si coating, and this is why the region E in Figure 8c is brighter than surrounding areas. Even so, the GA, GI and Al–Si coatings in region E are all complete without exposing the substrate, proving that the three coatings can provide the good lubrication for the steel sheet in plane sliding.

**Figure 10:**
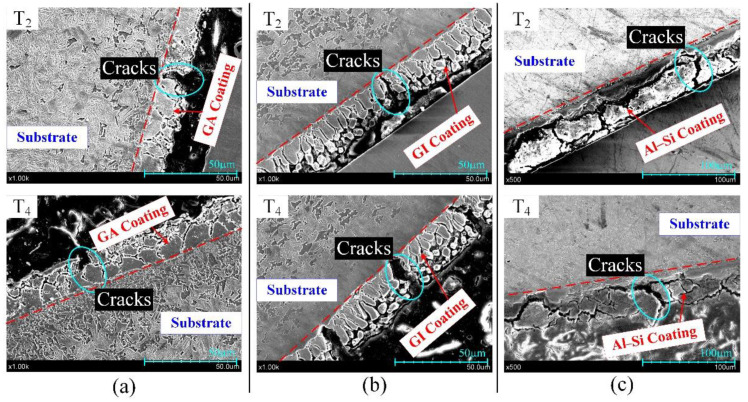
The microstructure of coatings at T2 and T4: (**a**) GA coating; (**b**) GI coating; (**c**) Al–Si coating.

The microstructures of GA, GI and Al–Si coatings at the round corners, where they suffer bigger deformation, were also observed by SEM and are listed in Figure 10. As seen in Figure 10a, there are some bigger cracks in GA coatings and their width is about 5 μm. The wide cracks originate from the coating surface and then vertically extend towards the substrate until stopping at the interface of coating and substrate. Obvious cracks with different sizes exist in the GI coating in Figure 10b. Different as those in GA coating, the crakes in GI coating can penetrate through the coating and even into the substrate. The GA and GI coatings both belong to zinc-based coatings, but the density and size of the cracks in GI coating are both bigger than that in GA coatings. The Al–Si coatings in Figure 10c are both divided into many individual blocks by cracks, which extend from the coating surface until closing the interface between coating and substrate and then continue to extend parallel to the interface.

The difference between the cracks in GA, GI and Al–Si coatings comes from the different coating compositions in the forming process. Because of the lower Fe content in the GI coating, the heated GI coating will contain a certain amount of liquid Zn apart from the α-Fe (Zn) phases. The existence of liquid Zn will easily cause more and bigger cracks in the GI coating when under tensile stress because of the LMIE phenomenon [28]. After heating under 930 °C for 4 min, the Al–Si coating will present as brittle because it mainly consists of brittle phases, such as Fe_2_SiAl_2_, Fe_5_SiAl_4_ and FeAl_2_ [7]. The phase in Al–Si coating nearing the interface is the solid solution α-Fe (Al + Si) phase, which could prevent the propagation of cracks because of its good plasticity [8].

### 3.3. The Quenching Performance of Different Coatings

**Figure 11:**
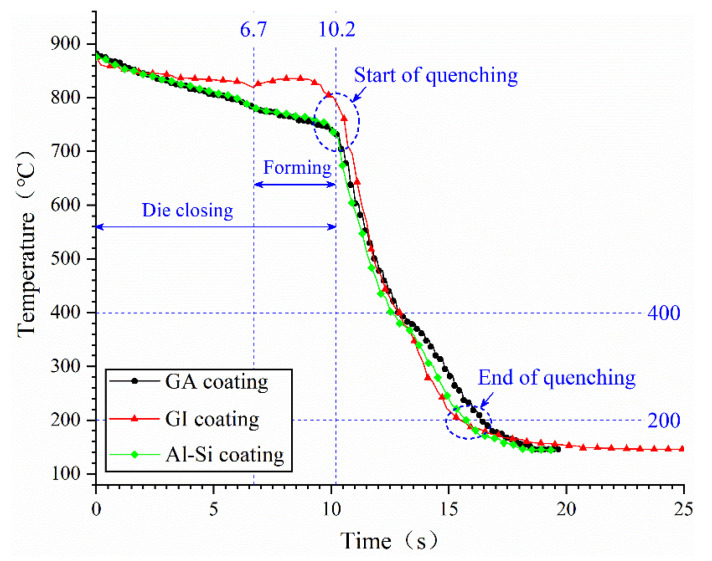
The temperature variation curves of the three coatings under the same forming-quenching process.

The temperature variation curves of samples with three coatings, detected at T_7_ in Figure 4, were compared in Figure 11. It took about 3.5 s to form the heated sheet and the initial forming temperature was about 800 °C. The quenching process started at about 730~800 °C and finished at 200 °C, taking about 5.4~6 s. The temperature variation curves in Figure 11 are analyzed in Table 3. When under the same forming process, there is no significant difference between the temperature variation curves. The cooling of the GI coated plate is the fastest and the cooling of the GA-coated plate is the slowest. The cooling speed of the plate in this paper depends on the heat conductivity of coating when taking the same experiment device and process. Therefore, the thermal conductivity of the GI coating is better than Al–Si and GA coatings.

**Figure 12:**
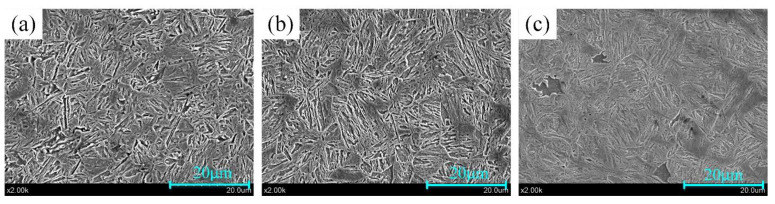
The substrate microstructure of samples at T_7_: (**a**) GA coated sample; (**b**) GI coated sample; (**c**) Al–Si coated sample.

As known from Table 3, the cooling speeds of GA, GI and Al–Si coated samples are all beyond 27 °C/s [29], so the martensitic transformation could be realized, which can be also be verified by the microstructure photos in Figure 12. In Figure 12, all the substrates under GA, GI and Al–Si coatings are full of martensite. Thus, the differences between the content of GA, GI and Al–Si coatings will not significantly affect the quenching performance of samples.

### 3.4. Summary and Outlook

The anti-oxidation property, forming and quenching performances of GA, GI and Al–Si coatings have been compared and their differences have also been analyzed from the microstructure. Nevertheless, the properties of coatings finally depends on the elementary compositions of coatings, and the relationship between them are still unclear. Thus, more experimental analyses should be conducted in the future, which is useful for improving the properties of boron steel sheet coatings in hot stamping processes.

## 4. Conclusions

The hot stamping performance of U-type parts covered with GA, GI and Al–Si coatings were researched in this paper and the following conclusions were obtained:

(1) When hearted at 930 °C for 3~4 min, GA, GI and Al–Si coatings will present as different colors because of the different consistency of oxides at the coating surface. There is no bare area of the substrate, showing that all the GA, GI and Al–Si coatings could provide the antioxidant protection of the substrate.
(2) The heated GA, GI and Al–Si coatings could all act as the lubricant in hot stamping to avoid friction damage between sheet substrate and die surface. Unlike Al–Si coatings, there are many micro cracks in worn GA and GI coatings, resulting in the macro scratches in the worn region of GA and GI coated samples.
(3) When the under similar deformation, more and larger cracks will occur in the GI coating at the round corners than in GA and Al–Si coatings, and will even damage the substrate because of LMIE phenomenon. The Al–Si coating can provide the best substrate protection because of the ductile layer between Al–Si coating and substrate.
(4) There is no significant difference between the temperature variation curves of GA, GI and Al–Si coated samples in quenching. The thermal conductivity of the GI coating is slightly better than the Al–Si and GA coatings.

## Figures and Tables

**Table 1:** The chemical compositions of boron steel B1500HS.

Element	C	Si	Mn	P	S	B	Cr	Ni	Al	Fe
Percentage (wt.%)	0.23	0.25	1.35	0.02	0.002	0.0026	0.17	0.016	0.046	Bal.

**Table 2:** The parameters of size and heating process of coated sheets.

Coatings	Size (mm × mm)	Thickness (mm)	Coating Thickness (μm)	Heating Temperature (°C)	Holding Time (min)
Al–Si	290 × 200	1.4	≈25	930	4
GA			≈13		3
GI			≈13		3

**Table 3:** The parameters of temperature variation curves in Figure 11.

Coatings	Holding Pressure (ton)	Quenching Start Temperature (°C)	Quenching End Temperature (°C)	Quenching Time (s)	Average Quenching Speed (°C/s)
GA	100	730	200	6.2	85.48
GI		801		5.3	113.4
Al–Si		723		5.46	95.79

## Data Availability

Data available on request due to restrictions eg privacy or ethical. The data presented in this study are available on request from the corresponding author.
